# ﻿A new species of the Neotropical genus *Smilidarnis* Andrade (Hemiptera, Membracidae), with a new country record for the genus

**DOI:** 10.3897/zookeys.1219.131379

**Published:** 2024-11-28

**Authors:** Stuart H. McKamey

**Affiliations:** 1 Systematic Entomology Laboratory, Agricultural Research Service, U.S. Department of Agriculture, c/o National Museum of Natural History, P.O. Box 37012, Washington, D.C. 20013, USA National Museum of Natural History Washington United States of America

**Keywords:** Bolivia, French Guiana, Neotropical, spelling fixation

## Abstract

*Smilidarnis* is **fixed as the correct original spelling**. *Smilidarnissimilifasciatus*, **sp. nov.**, from Bolivia and French Guiana, closely resembles *S.fasciatus* Andrade in being brightly colored but differs in the metathoracic tibial chaetotaxy, the male pygofer, first anal segment, aedeagus, and color pattern. In this new species, which is larger than *S.fasciatus*, females are larger than males. Photographs of the male and female adults and genitalia of the new species are provided. Bolivia is a new country record for the genus. A key to all species is provided.

## ﻿Introduction

Membracid treehoppers are famous for their hyperdeveloped pronotum, which is usually expanded to cover much of the body or projected upwards and posteriorly over it. Many species are cryptically colored yet others exhibit the classic aposematic coloration, such as bright red, orange, or yellow, sometimes over a black ground color, advertising their presence to potential predators (Joron 2009).

The higher taxa of Membracidae are largely based on forewing venation, leg chaetotaxy, and coverage of scutellum and wings by the pronotum. Andrade (1989) established the genus *Smilidarnis* for two new species without placing the genus to higher taxon because they display features of both Smiliinae and Darninae. His diagnosis was expanded slightly by McKamey (2023) to accommodate the new variation found among three new species. Specifically, Andrade reported metathoracic tibial cucullate rows II and III double and, by implication, row I single, but *S.duocornus* McKamey has row I absent and *S.robustus* McKamey has rows I and III double and row II absent. In addition, two of the new species described by McKamey (2023) have suprahumeral horns. *Smilidarnis* is still unplaced within the family. Features indicating placement in Smiliinae include Andrade’s species having metathoracic tibial chaetotaxy matching that of Ceresini, and in *S.concolor* Andrade the forewing veins R and M are confluent near its apex, which is a diagnostic feature of Smiliinae (Deitz 1975). *Smilidarnis* also has features of Darninae: some *Smilidarnis* have the metathoracic tibia with setal row I or II absent (Hemikypthini and some Darnini lacking row I, some Hemikypthini lacking row II); and R_4+5_ and M_1+2_ are separate throughout (Fig. 2), which occurs in all Darninae and no Smiliinae. Consequently, leg chaetotaxy and forewing venation do not provide evidence resolving the relationship of *Smilidarnis* to other treehoppers. Based on morphology, the phylogenetic estimate by Dietrich et al. (2001) placed Smilidarnis as the sister-group to seven tribes of Smiliinae. A more recent, unpublished molecular phylogeny by Evangelista et al. (2017) placed *Smilidarnis* as the sister group of Ceresini (Smiliinae). No other phylogenetic estimates have included *Smilidarnis*.

**Figures 1–6.**
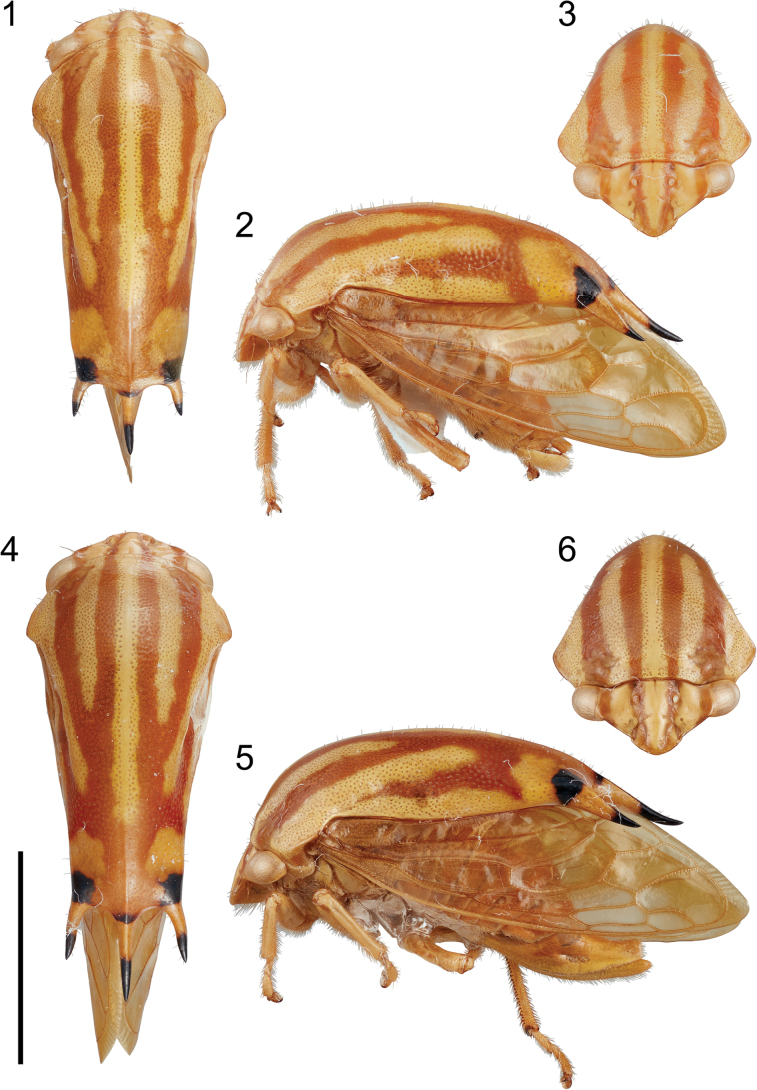
*Smilidarnissimilifasciatus* sp. nov. **1–3** male habitus in dorsal. lateral, and anterior views, respectively **4–6** female habitus in dorsal, lateral, and anterior views, respectively. Scale bar: 5 mm.

Until now, *S.fasciatus*Andrade (1989) was the only brightly colored species in the genus. It is recorded only from French Guiana and Peru. Lapèze (2021) collected three specimens of *S.fasciatus* at lights in two localities in French Guiana. He noted that in contrast to most membracids, the female was smaller than the male. The female habitus photographs by Lapèze (2021, p. 118) match the line drawing by Andrade (1989; Figs 1, 2) in every respect. A newly discovered species closely allied to *S.fasciatus*, and as brightly colored, is represented by a series of six specimens of males and females, from Bolivia, which were also collected at lights. The series of the new species provides an opportunity to investigate male vs. female variation in size, morphology, and coloration. The color pattern in the photograph by Sakakibara of a specimen from French Guiana (Deitz and Wallace 2010; distribution by Dr. A. M. Sakakibara, pers. comm.) is consistent with that of the new species.

## ﻿Materials and methods

In quoting labels, quotation marks separate labels and a vertical line separates lines on a label.

Terminology for general morphology, forewing venation (except crossvein s), and leg chaetotaxy follows Deitz (1975) except the gonoplac for the female genitalia, which follows Mejdalani (1998).

The abdomen was detached, macerated in a 10% KOH solution at room temperature for 72 hr, bathed in water, then in acetic acid to stop the reaction. After dissection, structures were stored in a glass microvial containing glycerin and pinned beneath the specimen.

A Leica MZ12 stereomicroscope was used to examine structures. All measurements were taken directly from the metadata of images from all specimens.

Images were taken with a Canon 5Dsr camera with an adjustable 65mm lens. Photos were taken using Capture One Pro version 10.1.2, 64 bit, build 10.1.2.23 imaging software, aided by CamLift version 2.9.7.1. The specimen was lit using two adjustable Dynalite MH2050 RoadMax flash heads, each attached to a Manfrotto 244 arm. The light was diffused using a lampshade-style cone of translucent paper between the specimen and light sources. After individual focal planes were photographed, they were compiled into a single, composite image using Zerene Stacker - USDA SI-SEL Lab Bk imaging system, version 1.04, build T201706041920. Stacked images were enhanced and edited in Adobe Photoshop CSS Extended version 12.0. The scale bars were generated through Photoshop directly from the metadata of the photos.

All specimens are deposited in the U.S. National Museum of Natural History, Smithsonian Institution, Washington, DC (**USNM**).

## ﻿Results

*Smilidarnis*Andrade (1989) is here fixed as the correct original spelling in accordance with ICZN Article 32.5 (International Commission on Zoological Nomenclature 1999); it was misspelled *Smiliodarnis* in the discussion. The Bolivian specimens of the new species represents a new country record for the genus.

### ﻿Key to species

**Table:** 

1	Pronotum with pair of stout suprahumeral spines	**2**
–	Pronotum without suprahumeral spines	**3**
2	Breadth across suprahumeral spines distinctly greater than breadth across posterior lateral spines (McKamey 2023; Fig. 2)	***S.duocornus* McKamey**
–	Breadth across suprahumeral spines subequal, slightly less than breadth across posterior lateral spines (McKamey 2023, fig. 14)	***S.robustus* McKamey**
3	Pronotum posteriorly with tips but not bases of lateral spines black; forewing with veins R and M distally fused then separated preapically	***S.concolor* Andrade**
–	Pronotum posteriorly with bases of lateral spines black; forewing with veins R and M not fused at any point	**4**
4	Pronotum with central apical spine pale throughout; head vertex with ventrolateral margins and frontoclypeus forming evenly convex curve (McKamey 2023; Fig. 9)	***S.erwini* McKamey**
–	Pronotum with central apical spine black in distal third; head vertex with ventrolateral margins straight and frontoclypeus forming an angle (Fig. 3)	**5**
5	Pronotum with lateral margin having an isolated yellow patch	***S.fasciatus* Andrade**
–	Pronotum with most lateral yellow band bifurcate posteriorly, lower arm running along almost entirety of lateral margin (Figs 2, 5)	***S.similifasciatus* sp. nov.**

#### ﻿ Smilidarnis similifasciatus sp. nov.

Taxon classificationAnimaliaHemipteraMembracidae

F632CF68-B582-51BB-8367-AEAA7215B197

https://zoobank.org/FC4868AC-C340-4292-B7E5-778D81965EC2

##### Diagnosis.

Pronotum without suprahumeral spines; brightly colored with orange, yellow, and black; most lateral yellow band bifurcate posteriorly, lower arm running along almost entirety of lateral margin.

##### Distribution.

Bolivia (**new country record for genus**), French Guiana.

##### Description.

**Figures 7–13.**
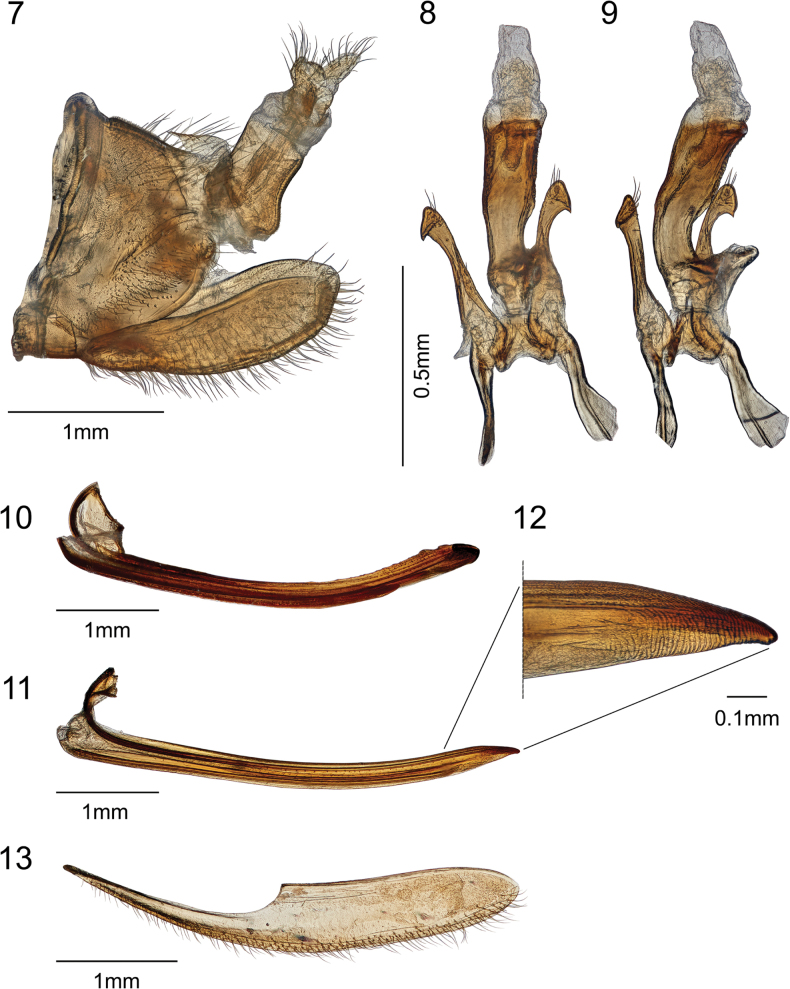
*Smilidarnissimilifasciatus* sp. nov. **7** male pygofer, lateral view **8, 9** aedeagus and styles in dorsanterior and oblique view, respectively **10** female valvula I **11, 12** female valvula II **13** gonoplac.

Dimensions (mm). Pronotal length ♀10.3–10.8, ♂9.8–9.9; total length including wings in repose ♀12.1–12.6, ♂11.5; width between humeral angles ♀4.7–4.8, ♂4.5–4.7; head width including eyes ♀3.9–4.0, ♂3.7–3.9, head width excluding eyes ♀2.7–2.8, ♂2.5–2.7; head height ♀1.7–1.8, ♂1.7–1.8; distance between apices of posterolateral spine apices ♀2.6–2.9, ♂2.4–2.6. ***Head*** (Figs 3, 6). Vertex inclined forward, wider than tall, glabrous, vertical dark lines including ocelli depressed; dorsal margin weakly convex, lateral margins straight; ocelli circular, on imaginary middle line between eyes; distance from outer margin of ocellus to eye 1.13 × distance between inner margins of ocelli; frontoclypeal sutures prominent; frontoclypeus in anterior view ventrally convex, in line with vertex ventrolateral margins. ***Pronotum*** (Figs 1–6). Suprahumeral spines absent; weakly elevated immediately behind humeral angles; distally with 3 spines, all directed ventroposteriorly, lateral pair directed weakly laterally; lateral spines reaching to leveI above r-m crossvein, central spine surpassing forewing vein M_3+4_. ***Legs*.** All femora lacking cucullate setae and spines; metathoracic tibia row I cucullate setae basally in single row, distally double, row II in single row, sparse and with minute basal hoods, row III cucullate setae in single row. ***Female terminalia*.** Sternite VII deeply emarginate medially; pygofer long; valvula I (Fig. 10) long, with one weak, wide dorsal process distally, apex rounded; valvula II (Figs 11, 12) in lateral view narrow and lanceolate with dorsal margin linear, lacking dorsal dentae or sinuations, apex subacute, ventrally striate; gonoplac (Fig. 13) long, distally wide, apex rounded, bearing macrosetae along entire ventral margin. ***Male terminalia*** (Figs 7–9). Abdominal segment X (1^st^ anal segment) with basaoventral lobe (Fig. 7); pygofer (Fig. 7) including lateral plate subquadrate, lateral plate with weak dorsal lobe, ovate, pilose throughout, subgenital plate fused basally, its sides subparallel, slightly narrowing distally, length in ventral view about 2.3 × width; style (Figs 8, 9) recurved with short acute apex bearing 4 setae preapically; aedeagus (Figs 8, 9) U-shaped in lateral view, shaft subparallel in anterior and lateral views, lacking dentae or other texture on distoanterior face but with minute dentae along lateral margins (Fig. 9); gonopore dorsal. ***Color*.** Male and female alike in color; pronotal color orange with 5 yellow longitudinal bands, medial and most lateral band nearly extending to large yellow spot in posterior fifth, most lateral longitudinal band bifurcate at mid length into 2 solid branches, 1 running along lateral margin, and black at bases and apices of posterior spines; head orange with 3 vertical yellow bands connecting to medial and closest yellow bands of pronotum; forewing anteriorly clear, posteriorly with amber tint.

##### Material examined.

*Smilidarnissimilifasciatus*, ***holotype*** • ♂ with labels “BOLIVIA, Dept. Santa Cruz | Prov. Andres Ibañez, Potreillos del Guenda, 17°40.27’ S, | 63°27.45’ W, 370 m, 5 Dec 2008 | T. J. Henry, at MV/black light” and red label “HOLOTYPE | Smilidarnis | similifasciatus | McKamey” (USNM). ***Paratypes*** • 4 ♀, 1 ♂, all same data as holotype but with blue paratype labels (USNM).

##### Etymology.

The specific name is a masculine Latin adjective based on a combination of “*similis*” (similar to) and the allied species “*fasciatus*”.

##### Discussion.

*Smilidarnisfasciatus* and *S.similifasciatus* key out in the same couplet because they are superficially similar. Nevertheless, they differ in several respects. Regarding coloration, in the new species all yellow stripes are longer in both males and females than those of *S.fasciatus*. Regarding metathoracic tibia, cucullate setal rows are double rows in *S.fasciatus* but single rows in the new species (row I distally double). Regarding terminalia, in the new species the pygofer and lateral plate combined is more quadrate than in *S.fasciatus*, the lateral plate itself is ovate (vs. subquadrate in *S.fasciatus*), segment X has a basoventral lobe present (vs. absent in *S.fasciatus*), and the aedeagal dentae are lateral (vs. on the face of the shaft in *S.fasciatus*).

The female habitus photographs by Lapèze (2021) match the illustration of *S.fasciatus* by Andrade (1989), while the photograph by Sakakibara (Deitz and Wallace 2010) corresponds to *S.similifasciatus*. Both species, therefore, are recorded from French Guiana. The *S.similifasciatus* male terminalia is most similar to that of *S.erwini*McKamey (2023) in that both have abdominal segment X bearing a basoventral lobe and the aedeagus lacking dentae or other texture on the dorsoanterior face of the shaft. The female genitalia of *S.fasciatus* has not been described so no comparison with that species is possible here, and only the second valvulae of *S.concolor* has been illustrated and described. The female genitalia of *S.similifasciatus* resembles those of *S.duocornus*, *S.robustus* and the second valvulae of *S.concolor*, in having the first valvulae long and narrow with poorly developed dentae distally, the second valvulae lanceolate, and the gonoplac distally wide with long setae ventrally.

Although the Bolivian males of the new species are slightly smaller than the lengths given by Andrade (1989) and Lapèze (2021) for *S.fasciatus*, in *S.similifasciatus* the four females are all larger than both males in every respect except head height, in contrast to Lapèze’s (2021) opposite finding about the relative lengths of genders of *S.fasciatus* in French Guiana. Also, the females of *S.similifasciatus* are longer than those of *S.fasciatus* as reported by Lapèze (2021).

## Supplementary Material

XML Treatment for Smilidarnis similifasciatus

## ﻿Additional information

### Author ORCIDs

Stuart H. McKamey https://orcid.org/0000-0002-9617-0594

### Data availability

All of the data that support the findings of this study are available in the main text.
